# Transcriptional landscape of Kaposi sarcoma tumors identifies unique immunologic signatures and key determinants of angiogenesis

**DOI:** 10.1186/s12967-023-04517-5

**Published:** 2023-09-22

**Authors:** Ramya Ramaswami, Takanobu Tagawa, Guruswamy Mahesh, Anna Serquina, Vishal Koparde, Kathryn Lurain, Sarah Dremel, Xiaofan Li, Ameera Mungale, Alex Beran, Zoe Weaver Ohler, Laura Bassel, Andrew Warner, Ralph Mangusan, Anaida Widell, Irene Ekwede, Laurie T. Krug, Thomas S. Uldrick, Robert Yarchoan, Joseph M. Ziegelbauer

**Affiliations:** 1 HIV and AIDS Malignancy Branch, National Cancer Institute, Bethesda, MD 20892 USA; 2 Frederick National Laboratory for Cancer Research, Frederick, MD USA

## Abstract

**Background:**

Kaposi sarcoma (KS) is a multicentric tumor caused by Kaposi sarcoma herpesvirus (KSHV) that leads to morbidity and mortality among people with HIV worldwide. KS commonly involves the skin but can occur in the gastrointestinal tract (GI) in severe cases.

**Methods:**

RNA sequencing was used to compare the cellular and KSHV gene expression signatures of skin and GI KS lesions in 44 paired samples from 19 participants with KS alone or with concurrent KSHV-associated diseases. Analyses of KSHV expression from KS lesions identified transcriptionally active areas of the viral genome.

**Results:**

The transcript of an essential viral lytic gene, ORF75, was detected in 91% of KS lesions. Analyses of host genes identified 370 differentially expressed genes (DEGs) unique to skin KS and 58 DEGs unique to GI KS lesions as compared to normal tissue. Interleukin (IL)-6 and IL-10 gene expression were higher in skin lesions as compared to normal skin but not in GI KS lesions. Twenty-six cellular genes were differentially expressed in both skin and GI KS tissues: these included *Fms-related tyrosine kinase 4* (*FLT4)*, encoding an angiogenic receptor, and *Stanniocalcin 1* (*STC1)*, a secreted glycoprotein*. FLT4* and *STC1* were further investigated in functional studies using primary lymphatic endothelial cells (LECs). In these models, KSHV infection of LECs led to increased tubule formation that was impaired upon knock-down of *STC1* or *FLT4*.

**Conclusions:**

This study of transcriptional profiling of KS tissue provides novel insights into the characteristics and pathogenesis of this unique virus-driven neoplasm.

**Supplementary Information:**

The online version contains supplementary material available at 10.1186/s12967-023-04517-5.

## Introduction

Kaposi sarcoma (KS) is caused by Kaposi sarcoma herpesvirus (KSHV, formally designated as human herpesvirus 8 (HHV8) [1]. In addition to KS, KSHV is the causative agent of other KSHV-associated diseases (KAD), such as primary effusion lymphoma (PEL), a form of multicentric Castleman disease (MCD), and more recently, KSHV inflammatory cytokine syndrome (KICS) [2–5]. These conditions can occur alone or concurrently in the same patient and have different treatments and prognoses [6, 7]. In the United States, KS is diagnosed predominantly among people living with HIV (PWH) and is known as epidemic KS. The incidence of HIV-associated KS has decreased with advances in HIV treatment [8] but KS frequently occurs among PWH in the US. Furthermore, KS is one of the most common cancers in areas in sub-Saharan Africa, where both endemic and epidemic KS are significant causes of morbidity and mortality [9].

Similar to other herpesviruses, KSHV infection generally exists either in a state of latency, in which a few of the viral genes are expressed and no infectious progeny are released, or lytic replication, in which most viral genes are expressed and infectious virions are produced and disseminate within and between hosts [10, 11]. Most spindle cells in KS lesions contain KSHV in its latent form, while a smaller number of cells express some KSHV lytic gene products [12, 13]. KS lesions are notable for the proliferation of pathognomonic abnormal spindle cells, mixed inflammatory infiltrates, and the formation of aberrant, leaky vascular channels [14, 15]. The spindle cells within KS lesions are infected with KSHV, and express vascular endothelial growth factor (VEGF) receptors and cytokines such as tumor necrosis factor-α and interleukin (IL)-6 [15–19]. KSHV, like other herpesviruses, is notable for its molecular piracy of genes homologous to cellular regulatory genes [20, 21] and its modulation of cellular survival, angiogenic and immune regulatory pathways [22, 23].

KS most commonly manifests as skin lesions but may also occur in visceral organs including the respiratory and gastrointestinal (GI) tracts in severe cases [24, 25]. Patients with GI KS often have gastrointestinal bleeding, anemia and weight loss. The presence of KS in the GI tract may indicate a diagnosis of a concurrent KAD, such as KICS, PEL or MCD. These concurrent KAD are associated with KSHV viremia and elevated circulating IL-6, which can result in systemic inflammation and multiorgan dysfunction [3, 26, 27]. However, there is limited information about the interaction between these clinical characteristics, inflammation, immunity and KSHV expression within KS tissues.

We sought to understand how host gene expression and viral transcripts are altered in KS as compared to normal tissue using matched patient samples. In this study of participants with well-annotated clinical characteristics, we also investigated both skin and GI KS to define differences in the viral and host transcripts between these sites of these lesions. We examined differences in the immune microenvironment by location of the KS lesion or by specific clinical characteristics, such as the presence of concurrent KAD. Specific cellular genes of interest that were identified from patient samples were further investigated using KSHV-infected lymphatic endothelial cells (LECs) infected to determine their pathological significance.

## Methods

### Patient cohort and specimen collection

Individuals with KS under the care of the HIV and AIDS Malignancy Branch at the National Cancer Institute were included. Clinical and HIV characteristics were obtained at the time of biopsy collection. KSHV viral load (VL) in peripheral blood mononuclear cells (PBMCs) was assessed by quantitative real-time polymerase chain reaction as previously described [28].

Participants had a 6 mm punch biopsy of cutaneous KS over the lower limb, with normal appearing skin obtained within the same limb. If participants had gastrointestinal symptoms, an endoscopy or colonoscopy was performed and the operator visually identified KS lesions that were biopsied. An adjacent area of normal mucosa was obtained at the same time. KS biopsies were divided, and a portion was sent to the laboratory of pathology for histological confirmation. Two samples (G4T and G5T) were biopsied as abnormal mucosa in patients with known skin KS but on analyses, these lesions did not have detection of KSHV LANA by immunohistochemistry staining. However, these lesions did have KSHV transcripts detected by RNA-sequencing and thus were included in these analyses. All participants were consented to protocols for tissue procurement (NCT00006518) and/or sequencing of KS and other KSHV-associated diseases (NCT03300830) to permit RNA sequencing of KS lesions for this study. Both protocols were approved by the NCI Institutional Review Board. All enrolled participants gave written informed consent in accordance with the Declaration of Helsinki.

### RNA sequencing and analysis

Tissues were stored in RNAlater and lysed with Trizol. Tissues were homogenized and extracted for total RNA with Direct-Zol Miniprep kit (Zymo). Ribosomal RNA was removed and sequencing libraries were prepared using Illumina TruSeq Stranded / NEBnext Ultra Low Input Total RNA Library Prep and paired-end sequencing. The reads from the FASTQ files (reads are generated by Illumina HiSeq4000) were trimmed of Illumina adapters using *cutadapt* [29] with default parameter except for –q 10 and –minimum-length 25. We generated a ‘combined genome reference’ by catenating the human reference genome (GRCh38) and KSHV reference genome (NC009333). The reads (average 45.9 million reads per sample) were aligned to the combined genome reference using STAR (version 2.7.8a) [30]. The transcriptome bam files created by STAR were used to estimate the read counts and Transcript per million (TPM) values at the gene level using RSEM v1.3.2 [31]. Annotations from GENCODE v30 for GRCh38 were combined with the KSHV gene annotations from NCBI while running STAR and RSEM.

To find differentially expressed genes (DEGs) between tumor and normal samples, pairwise comparison was carried out using DESeq2 [32]. DEGs were selected for the further study based on | log2FoldChange |> = 2 and adjusted p-value <  = 0.05. TPM values were used to generate scatterplot. All the plots were generated using R package ggplot2. The log2 foldchange values were considered for the jitterplot by comparing tumor vs normal using edgeR program [33]. The TPM values of viral genes were used to generate the heatmap using ComplexHeatmap package [34]. Raw and processed data are available on NCBI GEO accession number GSE241095.

### Staining for proteins and RNA transcripts in tissue sections

Staining for LANA (Leica mouse monoclonal PA0050 ready-to-use) and CD31 (Abcam rabbit polyclonal ab32457; 1:200 dilution) was performed on a fully automated BondMax autostainer (Leica) and detected with DAB using the HRP Polymer Refine Detection Kit (Leica) and hematoxylin counterstaining. FFPE sections of 5 µm were prepared from fixed tumor samples that were deparaffinized and rehydrated with a series of ethanol washes to deionized water. Sections were subjected to citrate (CD31) or EDTA-based (LANA) antigen retrieval for 20 min prior to immunostaining. Slides were incubated with primary antibody for 15 min (LANA) and 30 min (CD31). All slides were scanned and analyzed on an Aperio ImageScope scanner (Leica) and reviewed by pathologist L. Bassel. Human herpesvirus 8 ORF75 and viral IL-6 expression was detected by RNA in situ hybridization. 5 um FFPE tissue sections were hybridized with RNAscope 2.5 LS Probes V-HHV8-ORF75 (ACD, Cat# 562058) and V-HHV8-K2-C3 (ACD, Cat# 897938-C3) using the RNAscope® LS Multiplex Fluorescent Assay (ACD, Cat# 322800) with a Bond RX auto-stainer (Leica Biosystems) with a tissue pretreatment of 15 min at 90 °C with Bond Epitope Retrieval Solution 2 (Leica Biosystems), 15 min of Protease III (ACD) at 40 °C, and 1:750 dilution of TSA Plus-Cyanine 3 (AKOYA Biosciences, cat# NEL744001KT) and TSA Plus-Cyanine 5 (AKOYA Biosciences, cat# NEL745001KT), respectively. The RNAscope® 3-plex LS Multiplex Negative Control Probe (Bacillus subtilis dihydrodipicolinate reductase (dapB) gene in channels C1, C2, and C3, Cat# 320878) was used as a negative control. The RNAscope® LS 2.5 3-plex Positive Control Probe- Hs was used as a technical control to ensure the RNA quality of tissue sections was suitable for staining. Slides were digitally imaged using an Aperio ScanScope FL Scanner (Leica Biosystems).

### Cell culture, reagents, nucleofection, and KSHV infection

Human dermal lymphatic endothelial cells (HDLECs) were obtained from Promocell and passaged in EGM2 medium (Lonza) for up to 5 passages, with passages 3 to 5 used for experiments. ON-TARGETplus nontargeting control siRNA and ON-TARGETplus SMARTpool siRNA targeting *STC1* and *FLT4* were obtained from Dharmacon/Horizon Discovery. iSLK-BAC16 cells were induced with 1 ug/mL Doxycycline and 1 mM Sodium Butyrate for 3 days. Cell debris was removed from the supernatant fraction by centrifuging at 2000 × *g* 4 oC for 10 min and filtering with a 0.45 PES membrane. Virus was concentrated after a 16,000 × *g* 4 oC 24 h spin and resuspended in a low volume of EGM2 media (approx. 1000-fold concentration). To assess viral infectivity, LEC were infected with serial dilutions of BAC16 stock and assessed using CytoFlex S (Beckman Coulter) for GFP + cells at 3 days post infection. BAC16 contains a constitutively expressed GFP gene within the viral genome. Based on these assays, BAC16 stock was used at a 1:60 dilution, resulting in 70% infection for LEC (MOI 1). HDLEC de novo infections were carried out using KSHV-BAC16 (concentrated by ultracentrifugation), diluted in EGM2 medium. Polybrene was added at 8 μg/ml. Control infection experiments were conducted with polybrene to test whether potential downstream events could be explained by a difference in viral entry. Uninfected samples were included as negative controls. After 16 h of incubation, cells were washed and overlaid fresh media. RNA was harvested and extracted using Direct-zol kit (Zymo) with DNAse treatment. Viral entry was measured by detecting GFP-positive cells using flow cytometry. Viral entry was also measured by treating cells with Proteinase K, then phenol–chloroform-isoamyl alcohol, and ethanol precipitation of DNA to measure intracellular viral DNA. Viral DNA was measured by qPCR. Viral particles were collected from conditioned media after filtering with 0.45 µm filter. Filtered material was digested with DNAse I and virion DNA was purified using Proteinase K, then phenol–chloroform–isoamyl alcohol, and ethanol precipitation of DNA.

### Measuring viral genomes

The cell fraction was isolated from infection models. Cell pellets were washed with 1 × PBS and lysed using 0.5% SDS, 400 µg/mL proteinase K, 100 mM NaCl. Samples were incubated at 37 °C for 12–18 h and heat inactivated for 30 min at 65 °C. DNA samples were serial diluted to 1:1000 and measured using qPCR with primers specific to KSHV ORF6 or human GAPDH. Standard curves were generated using purified genomic stocks (KSHV BAC16, human genome Promega #G1471). Absolute copy number of genomic stocks was determined using ddPCR. Values were plotted as follows:

$${\text{viral}}\;{\text{genomes}}/{\text{cell}} = \frac{{{\text{viral}}\;{\text{gene}}\;{\text{copy}}\;{\text{number}}}}{{{\text{host}}\;{\text{gene}}\;{\text{copy}}\;{\text{number}}/2}}$$

### Lytic reactivation of KSHV

Subconfluent monolayers of iSLK-BAC16 were induced with 1 ug/mL Doxycycline, 1 mM Sodium Butyrate in DMEM media supplemented with 2% Tet-approved FBS. 0 h time point was when induction media was added and cells were first placed at 37 °C to incubate.

### Quantitation of mRNAs and proteins

Quantitative reverse transcription-PCR (RT-qPCR) was performed using 200–500 ng RNA and random primers with an Applied Biosystems high-capacity cDNA reverse transcription kit. SYBR green assays (FastStart universal SYBR green master mix; Roche or Thunderbird SYBR qPCR master mix, Toyobo) and TaqMan assays (TaqMan Universal PCR master mix, no AmpErase UNG; STC1 Assay ID: Hs00174970_m1, FLT4 Assay ID: Hs01047677_m1, Applied Biosystems) were performed using the ABI StepOnePlus real-time PCR system (Applied Biosystems). Relative mRNA levels were computed using the threshold cycle (ΔΔCt) method with RPS13 (Assay ID: Hs01011487_g1, Applied Biosystems) as a reference gene. Secreted STC1 was measured from LEC supernatant using STC1 ELISA (Human Stanniocalcin 1 DuoSet ELISA DY2958, Ancillary Kit DY008B, R&D Systems) according to manufacturer’s protocol. Antibodies for Western blotting include: Human Stanniocalcin 1/STC-1 Antibody # MAB2958 (RND), α/β-Tubulin Antibody #2148 (Cell Signaling), VEGF Receptor 3 (D1J9Z). Rabbit mAb #33566 (Cat#33566S) (Cell Signaling), GAPDH mouse monoclonal antibody [6C5] #ab8245 (AbCam). Blots were scanned on an Odyssey scanner (Li-Cor).

### Tube formation assays

HDLEC cells were transfected with siRNAs, then infected with KSHV BAC16 at MOI of 0.25. At 1 dpi, cells were split to keep them sub confluent. At 2dpi, cells were harvested, counted, and seeded in duplicate at 0.3 million cells/mL in 96-well plate on Cultrex Ultimatrix Reduced Growth Factor Basement Membrane Extract (Bio-Techne #BME001-05) for tube formation assays using the thick gel method[35]. At 16 h post-seeding, brightfield microscope images were acquired and analyzed by ImageJ and Angiogenesis Analyzer [36].

### Statistical analyses

An analysis of the sample size and statistical power for RNA sequencing was calculated using the R package, RnaSeqSampleSize. To achieve an 80% chance of detecting an effect, a sample size of 11, would be required when assuming an average of 2000 reads per gene, 16,000 total genes detected, a minimum fold change of 4, an FDR of 0.05, and 200 differentially expressed genes. For genomic-level expression analysis, adjusted p-values were calculated using the Benjamini and Hochberg method. To determine the gene expression difference between each KS and respective matched normal tissue sample from each participant, cellular genes with > log2 fold change of 2.0 with an adjusted p-value < 0.05 were identified. For smaller sets of comparisons, Student’s *t* test was used. To determine the correlation between human and KSHV viral gene expression, Spearman correlation analysis was used.

## Results

### Participant HIV and KS characteristics

**Fig. 1:**
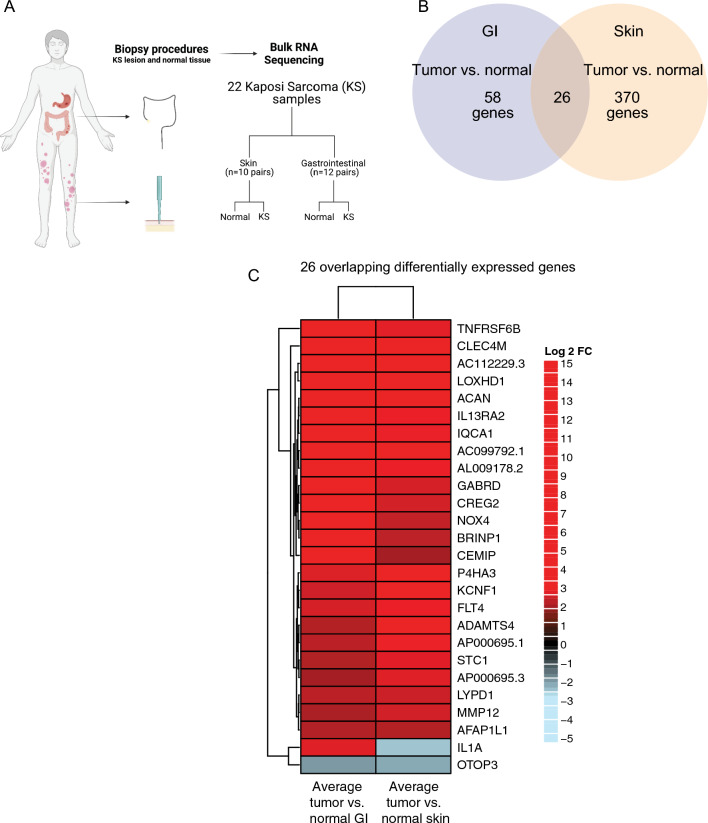
Sample types and RNA expression profiles. **A** Diagram of the normal and KS matched samples used in this study. Differentially expressed genes from GI (KS tumor versus normal) samples and differentially expressed genes from skin (tumor vs normal in green) samples. The differential gene expression cutoff of log2 Fold Change > or < 2.0 and padj. < 0.05 between tumor vs normal in both GI and skin samples was used. **B** Venn diagram shows unique and shared differentially expressed genes. **C** Heatmap showing shared differentially expressed genes using average Log_2_ fold change values

Nineteen participants contributed 22 KS samples with paired normal tissue, comprising 10 skin KS samples and 12 GI KS samples (Fig. 1A). Three participants provided both cutaneous and GI KS samples at the same timepoint. Seventeen participants (89%) had HIV-coinfection, 16 of whom were receiving antiretroviral therapy and had a median CD4^+^ T cell count of 38 cells/µL and HIV viral load (VL) of 443 copies/mL at the time of sample collection (Table 1). In addition to KS, 9 participants met criteria for KICS (diagnostic criteria noted in Additional file 1: Table S1), 1 participant had active concurrent MCD, 2 participants had PEL, and 2 participants had concurrent PEL and MCD. The median KSHV VL for all participants was 376.5 copies/10^6^ PBMCs and was higher for those with GI KS (1148 copies/10^6^ PBMCs). Participants with skin KS had a lower median HIV VL (74 copies/mL) as compared to those with GI KS (5134 copies/mL). CD4^+^ T cell counts were similar in participants with skin KS (38 cells/µL) and GI KS (36 cells/µL).

**Table 1:** Baseline characteristics of all participants at the time of biopsy collection and characteristics by skin and GI sample collection

	All participants (N = 19)	Skin KS participants* (N = 10)	GI KS participants* (N = 12)
Age (median in years, IQR)	37 (29, 46)	43 (34, 49)	30 (28.3, 36.1)
*Sex (n, %)*			
Cisgender Male	17 (89)	10 (100)	10 (83)
Cisgender Female	1 (5)		1 (8)
Transgender Female	1 (5)		1 (8)
*Race (n, %)*			
White	4 (21)	2 (20)	3 (25)
Black	10 (53)	4 (40)	7 (58)
Hispanic	5 (26)	4 (40)	2 (17)
*HIV characteristics*			
HIV co-infection (n, %)	17 (89)	9 (90)	11 (92)
Duration of HIV, median, (median in months, IQR)	95 (5, 147)	117 (5, 145)	97 (26, 107)
CD4 T-cell count, cells/µL (median, IQR)	38 (24, 100)	39 (19, 195)	36 (25, 85)
HIV viral load, copies/mL (median, IQR)	443 (57, 5134)	74 (39, 104)	5134 (552, 47,759)
On antiretroviral therapy (ART) at time of biopsy (n, % of HIV +)	16 (94)	10 (100)	11 (92)
*KS characteristics*			
Duration of KS diagnosis (median in months, IQR)	5 (0.9, 32)	5 (1, 38)	10 (1, 16)
Prior therapy for KS or other KAD (n, %)	7 (37)	3 (30)	6 (50)
*Concurrent KSHV-associated diseases (n, %)*			
None	5 (16)	5 (30)	3 (8)
KICS	9 (53)	3 (50)	6 (58)
MCD	1 (11)	1 (10)	
PEL	2 (11)	1 (10)	1 (8)
MCD and PEL	2 (11)	–	2 (17)
KSHV-VL, copies/10^6^ PBMCs (median, IQR)	377 (0, 1798)	90 (0, 728)	1148 (0, 2792)

^*^Includes 3 participants who provided both skin and GI samples

### Differences and overlap in differentially expressed genes in GI and skin KS lesions when compared with paired normal samples

First, gene expression patterns were examined in individual samples (Fig. 1A) using principal component analysis (PCA) (Additional file 1: Fig. S1A–C), which revealed that sample gene expression patterns were distinguished by KS lesions versus normal tissues. To measure overall levels of KSHV gene expression for each lesion, total transcript per million (TPM) values were calculated (Additional file 1: Fig. S1D).

Among approximately 17,000 mapped genes, there were 370 differentially expressed genes (DEGs) unique to skin KS and 58 DEGs unique to GI KS, and 26 common to both as compared to their respective normal tissues (Fig. 1B). One of the most enriched pathways in both skin and GI KS samples included those related to IL-6 signaling, HIFα signaling, granulocyte adhesion and diapedesis pathway (Additional file 1: Tables S2, S3). The B cell receptor signaling pathway was enriched in the skin KS samples, but not the GI KS samples. There were 26 DEGs that were common in both the GI and skin samples as compared to their matched normal samples (Fig. 1C). These included *Fms-related tyrosine kinase 4* (*FLT4)*, encoding an angiogenic receptor VEGFR3, and *Stanniocalcin 1* (*STC1)*, a secreted glycoprotein that has altered expression in several malignancies. *IL1A* levels were increased in GI KS but repressed in skin KS (Fig. 1C). Additional analyses identified a correlation between *IL1A* expression and total levels of viral expression in GI KS, which was not observed in skin samples (Additional file 1: Table S4). *OTOP3*, a gene associated with proton transport across the cell membrane, was the only gene repressed within all KS lesions as compared to the normal tissue.

### Genes associated with inflammatory response and cytokine dysregulation vary by KS location

**Fig. 2:**
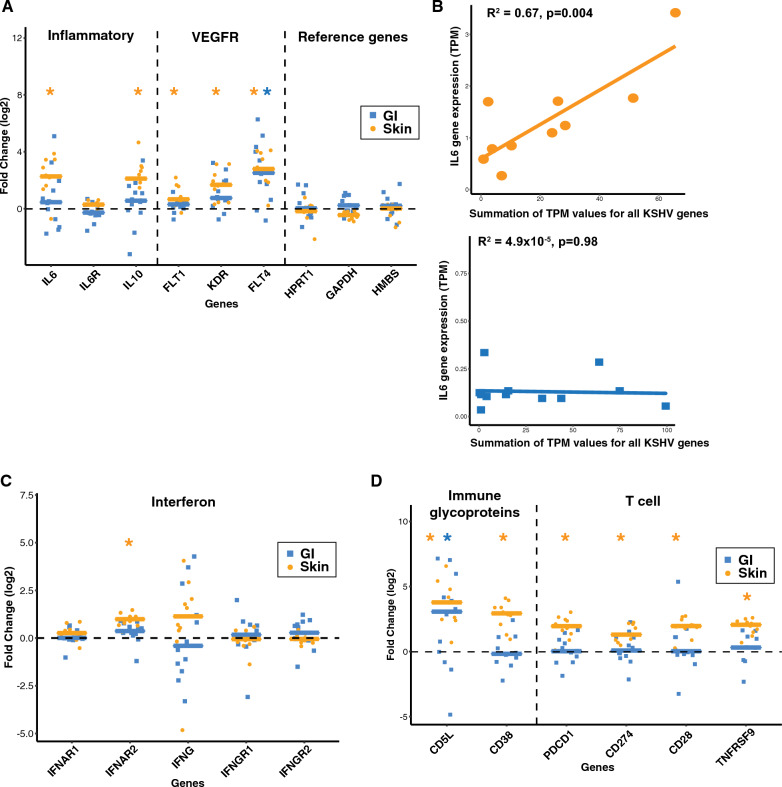
Selected genes associated with viral pathogenesis or immune responses are plotted. **A**, **C**, **D** Asterisks (blue* for GI and orange * for skin) represent statistically significant (Student’s t test, p < 0.05) genes, in individual GI (tumor vs normal, log2 Fold Change, blue squares) and skin (tumor vs normal, log2 Fold Change, orange dots) samples. **B** For only the KS tumor samples: transcripts per million (TPM) were collected for all KSHV genes on the horizontal axis and TPM for IL6 is shown on the vertical axis (orange for KS skin, blue for KS GI)

Genes associated with inflammatory responses were explored to study differential expression between KS lesions and normal tissue samples (Additional file 1: Fig. S2). As expected, housekeeping (*HPRT1, GAPDH, HMBS*) genes were not significantly altered in expression when comparing KS lesions to their normal matched tissue samples (Fig. 2A). *IL6* and *IL10* RNA levels were increased in skin KS (log2FC = 2.1, adj. p = 0.001; log2FC = 2.1, adj. p = 0.0009, respectively), but not significantly altered in GI KS lesions as compared to matched normal tissues. Furthermore, increased expression of *IL6* correlated with increased total KSHV gene expression in skin KS (R^2^ = 0.67, p = 0.004) but not in GI KS (Fig. 2B). We also examined genes associated with angiogenesis, as abnormal vascularity and aberrant angiogenesis are prominent features of KS lesions irrespective of their location [16, 37]. VEGF receptors 1, 2, and 3 are encoded by *FLT1*, *KDR*, and *FLT4*, respectively. All 3 VEGF receptor genes were upregulated in skin KS lesions and *FLT4* (*VEGFR3*) was increased in both skin and GI KS (log2FC = 2.8, adj. p = 0.00005; log2FC = 2.6, adj. p = 0.001, respectively (Fig. 2A)).

There was a wide range of expression changes in interferon-gamma (IFN-γ), which has been implicated to be a factor in combating KSHV infection [38]. Only *IFNAR2* showed consistent upregulation in skin KS samples (log2FC = 0.9, adj. p = 0.00001, Fig. 2C). In addition to interferon responses, changes in the expression of genes involved in the immune response were evaluated. *CD5L* expression, encoding a secreted protein that is predominantly released by macrophages, was increased in skin and GI KS lesions (log2FC = 3.7, adj. p = 0.00008; log2FC = 2.3, adj. p = 0.04997, Fig. 2D). CD38 is an activation marker expressed in a variety of immune cells, including natural killer cells, B lymphocytes, and CD4 + and CD8 + T lymphocytes. *CD38* expression was increased in skin KS (log2FC = 2.8, adj. p = 0.00002, Fig. 2D), but not in GI KS. PD1 signaling can suppress T-cell responses in chronic infection and cancer. *PDCD1* (*PD1*) and *CD274* (*PD-L1*) were modestly increased in skin KS, but not statistically significant in GI KS lesions.

### Expression markers in patients with KS alone as compared to KS with concurrent KAD

**Fig. 3:**
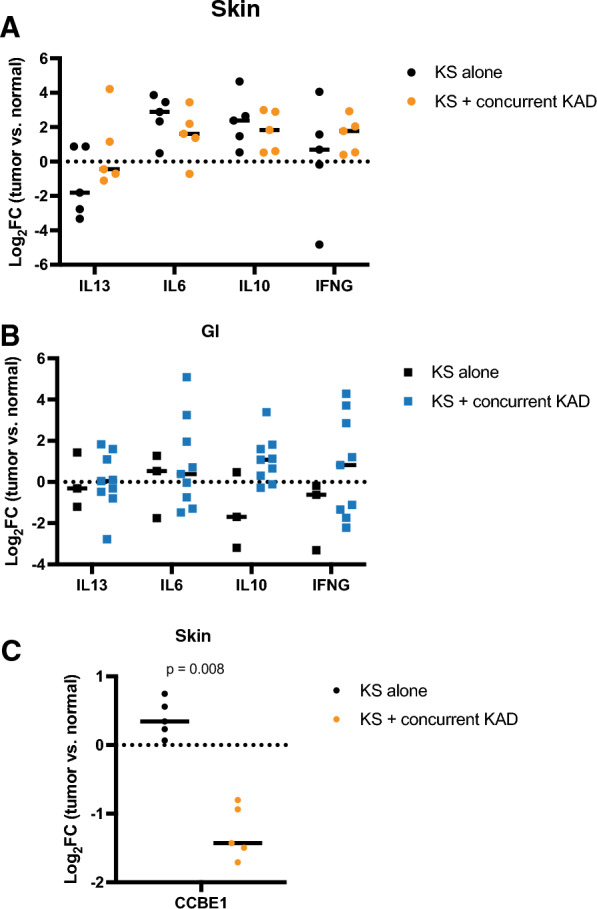
RNA expression samples were separated into samples from participants with only KS or with KS and additional KSHV associated diseases. **A**-**B**. Selected cytokines and expression changes between normal and tumor samples are shown. **C** The CCBE1 expression patterns in skin samples are shown

There were 5 participants who had KS alone and 14 participants had active concurrent KAD at the time of sample collection. The RNA expression patterns were integrated with clinical characteristics to identify new markers of concurrent KAD. Expression in KS samples (as compared to normal control samples) were separated by the presence of KS alone or KS with other KAD). There was no significant difference in *IL13, IL6, IL10*, or *IFNG* expression levels by a diagnosis of KS alone as compared to KS with concurrent KAD in the skin or GI KS lesions (Fig. 3A, B). The *collagen and calcium binding EGF domains 1* gene (*CCBE1*), a proposed tumor suppressor, was increased in skin KS samples from those with KS alone whereas the gene expression was lower among those with KS with concurrent KAD (log2FC 0.39 vs. -1.29 p = 0.008, Fig. 3C).

### KSHV gene expression in GI KS and skin KS lesions

**Fig. 4:**
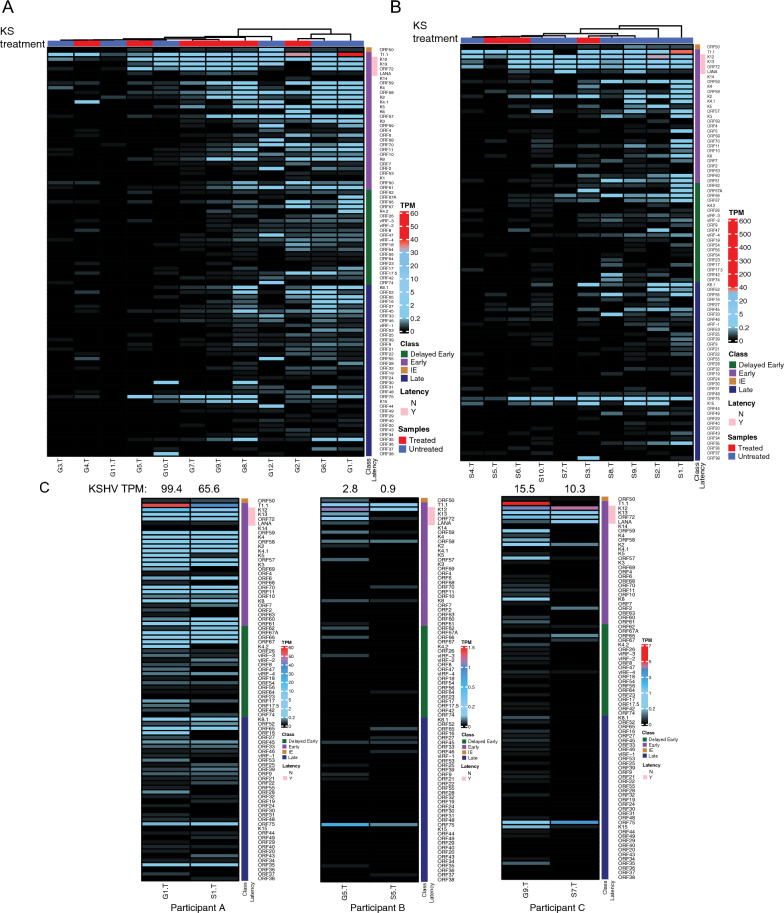
Heatmaps of KSHV gene expression KS tumors in GI (**A**) and skin (**B**) samples. Samples were either from participants untreated for KS (blue) or treated (red). Heatmap shows TPM expression from black (low) to medium (light blue) to high (red). ‘Class’ represent different stages of KSHV genes classification based on previous studies as Immediate Early (brown), Early (purple), Delayed Early (green), and Late (blue). The latency genes were represented as not latent (N -white) and latent (Y-pink). **C** KSHV gene expression heatmaps from the same matched participants. KSHV viral genes expression (Transcript per million (TPM) values (low-black, medium-blue, high-red) in GI and skin tumor samples from the same participants

We investigated whether canonical latent and lytic gene expression patterns were observed in KS biopsies. Among GI KS and skin KS, there was heterogeneity with respect to the KSHV expression patterns in the heatmap analyses (Fig. 4A, B). In general, three patterns were detected across multiple lesions. First, there was expression of T1.1/PAN, a lytic marker, with little expression of other lytic genes. Second, expression of canonical latency genes (LANA, ORF72, K12, K13) were found, as expected. Third, there was moderate to high expression (TPM greater than 0.1) of more than 10 KSHV genes in 6 of 12 GI lesions and 5 of 10 skin lesions. The analyses of KS lesions identified some unexpected trends in KSHV gene expression. ORF75 was noted in 91% of all KS lesions and high levels of ORF75 were observed when many other lytic genes were not expressed. This is unusual as ORF75 has been described as a late gene and only expressed in lytically-infected cells. Furthermore, the expression of ORF72/vCyclin D and LANA/ORF73 did not correlate, despite being adjacent genes in the latency locus.

Three participants provided both GI and skin KS lesions at the same timepoint. We noted similarities in the expression of ORF75 and K12, irrespective of location in these participants (Fig. 4C). Underlying clinical characteristics were considered for potential impact on KSHV gene expression in these individuals. All 3 participants had uncontrolled HIV at the time of sample collection, with a CD4^+^ T cell count of < 50 cells/µL. Patient A had no concurrent KSHV-associated diseases and had undetectable levels of circulating KSHV PBMC levels (Fig. 4C). This individual had uniform expression of lytic and latent KSHV genes in both samples. Among all 3 participants, high levels of transcription of ORF75 were noted in both skin and GI samples but few other late genes were expressed. In two of three participants (Patient A who had KS alone and Patient C who had KS and met criteria for KICS), there was higher T1.1/PAN expression in GI KS when compared to skin KS (Fig. 4C). There was also high expression of K2/viral IL6 in both skin and GI KS in same patients.

### KSHV and host gene expression in tissue sections

**Fig. 5:**
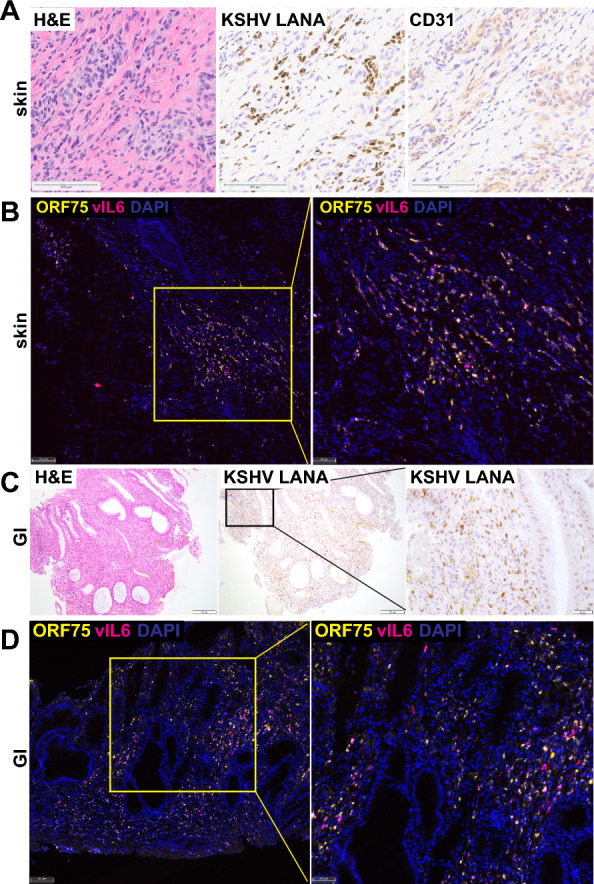
Expression of KSHV and human genes in KS lesions. **A** Sections from KS skin tumor sample S9T were stained with H&E for pathology or stained by immunohistochemistry for KSHV LANA protein or human endothelial marker CD31. Scale bars = 100 µm. **B** The same tissue sample was analyzed by RNA in situ hybridization with probes to the KSHV RNAs ORF75 and vIL6. Scale bars = 100 µm for left and 50 µm for right image. **C** KS GI tumor sample G2T was stained with H&E or for KSHV LANA protein. Scale bars = 100 µm for left two images and 20 µm for image on right. **D** KS GI tumor sample G2T was analyzed for expression of KSHV ORF75 and vIL6 RNAs. Scale bar = 100 µm for left image and 50 µm for right image

To determine whether ORF75, a marker of lytic replication was present in KS lesions, a randomly selected sample (noted in Fig. 4 as S9T) was studied using immunohistochemistry and RNA in situ hybridization assays. Robust protein expression of KSHV LANA protein, and the endothelial cell marker CD31 were observed in sequential sections of the same area of tissue by in situ hybridization (Fig. 5), findings consistent with KS pathology. Multiplex RNA in situ hybridizations were used to visualize viral transcript expression in a region verified to harbor KSHV infection based on LANA detection. KSHV ORF75 RNA was robustly detected in large clusters of cells that also expressed KSHV vIL6 RNA (Fig. 5B), in multiple areas of the biopsy. A similar analysis of KSHV LANA protein by IHC (Fig. 5C) and KSHV RNA by RNA in situ hybridization was conducted (Fig. 5D) with a GI KS tumor sample (G2T in Fig. 4). Expression of KSHV ORF75 and vIL6 RNA was detected throughout the GI KS lesion. These data suggests that the high levels of transcripts detected by RNA-seq are not limited to a small subpopulation of lytic cells and support the finding that transcription from the ORF75 genomic region is widespread in KS skin lesions.

### STC1 and FLT4 expression in de novo infections

**Fig. 6:**
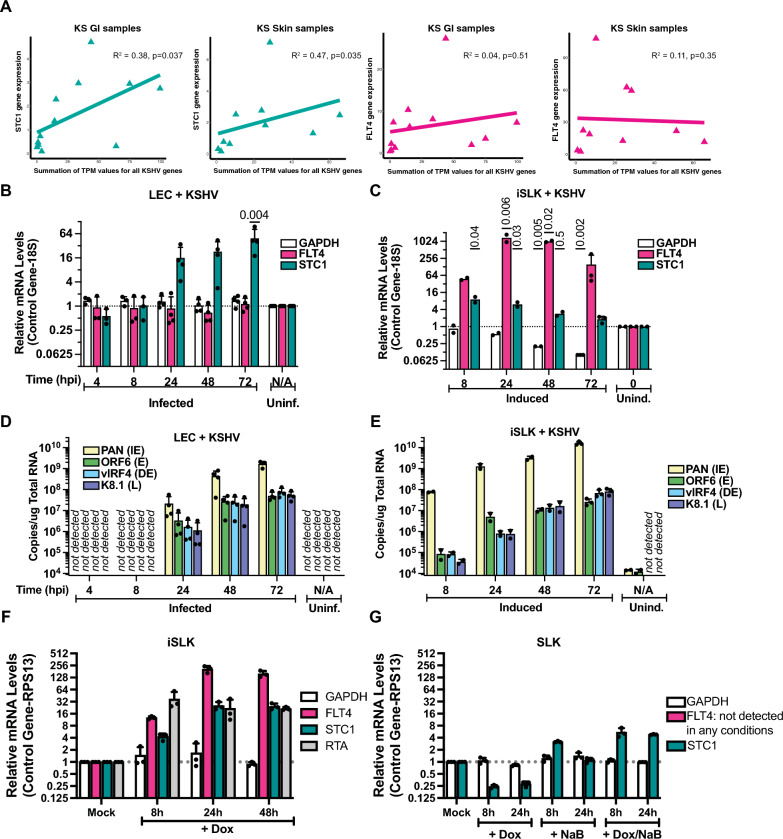
Gene expression in KS samples and cell culture assays. **A** Total KSHV transcript per million (TPM) values versus STC1 and FLT4 TPM values were plotted. Spearman correlation analysis was performed for KS tumor samples. **B** Human dermal lymphatic endothelial primary cells (LEC) were infected with KSHV and RNA expression was analyzed 4 to 72 h post-infection using qPCR. **C** KSHV-infected iSLK cells were lytically induced and RNA was harvested 8 to 72 h after induction. **D**, **E** Viral transcripts (immediate early, IE; early, E; delayed-early, DE; late, L) were measured using genomic standard curves. **F** iSLK cells without KSHV infection were treated with doxycycline (Dox) to induce RTA expression. **G** Control SLK cells lacking inducible RTA were treated with combinations of Dox and sodium butyrate (NaB)

Among the cellular genes that were upregulated in both GI and skin lesions as compared to normal tissues, STC1 and FLT4 were chosen for further investigation. Previous cell culture KSHV infections in primary human umbilical vein endothelial cells demonstrated an increase in STC1 expression in KSHV-infected cells [41]. STC1 was selected for further investigation due to the correlation between total KSHV gene expression and STC1 expression in GI and skin KS (Fig. 6A). Though FLT4 expression did not correlate with total KSHV gene expression in KS lesions (Fig. 6A), this gene has been upregulated in previous studies of KS [39–41]. We examined changes in STC1 and FLT4 upon KSHV infection in more controlled laboratory experiments with lymphatic endothelial cells (LECs). Within the LEC model, STC1 expression strongly increased with de novo KSHV infection (Fig. 6B). Secretion of STC1 protein was also increased at 2 dpi (Additional file 1: Fig. S3). In SLK cells, lytic induction of KSHV increased expression of FLT4 around 1000-fold (Fig. 6C), consistent with increased FLT4/VEGFR3 expression in TIME cells infected with KSHV [43]. At 48 and 72 h post-infection, KSHV PAN levels in LEC cells (Fig. 6D) was similar to levels in lytically-induced iSLK cells (Fig. 6E). Using control iSLK cells that were not infected with KSHV, it appeared that induced expression of the RTA transgene was sufficient to induce FLT4 RNA expression (Fig. 6F). The potential transcriptional regulation of STC1 was less clear since doxycycline and sodium butyrate treatment without an RTA transgene present in control SLK cells showed a modest induction of STC1 RNA expression (Fig. 6G). Overall, cell culture infections corroborated observations in GI and skin KS that showed an increase in expression of STC1 and FLT4 with KSHV infection or reactivation.

### Effects of STC1 and FLT4 on infection and angiogenesis in LECs

**Fig. 7:**
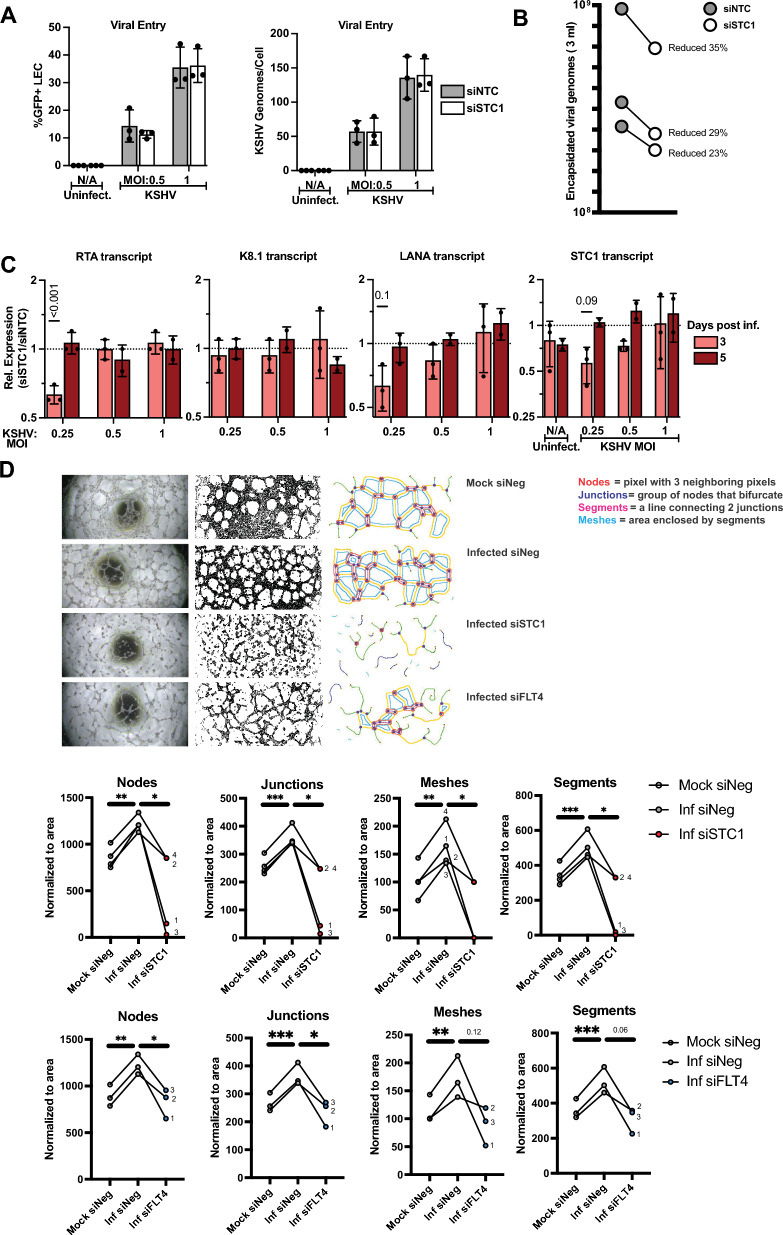
Repression of STC1 and FLT4 in primary human dermal lymphatic endothelial cells. **A** Cells were transfected with siRNA non-targeting control (siNTC) or siRNAs targeting STC1 (siSTC1). One day post-transfection, cells were infected with KSHV strain BAC16 (contains a GFP reporter). The percentage of GFP-positive cells was determined by flow cytometry (left). DNA harvested by cells was used in qPCR assays to measure viral genomes per cell at 1 day post-infection. **B** New viral particles were measured from conditioned media from cells infected and transfected. Samples were collected 5 days after infection. Each line represents a separate biological replicate. **C** RNA was purified from cells after transfection and infection and KSHV transcript levels were compared between siSTC1-transfected cells and siNTC cells. **D** Endothelial cells were transfected with siRNAs, infected (MOI = 0.25), then seeded on basement membrane extract at 2 dpi, and imaged by brightfield microscopy at 3 dpi. Tubule formation (Nodes, Junctions, Meshes, Segments) was assessed by image analysis software. Paired t-test was performed for statistical significance (*p < 0.05, **p < 0.01, ***p < 0.001)

Small interfering RNAs (siRNAs) were used to assess the impact of STC1 and FLT4 expression on viral infection and angiogenesis. Primary LECs were transfected with an siRNA control (siNTC) or siRNAs targeting *STC1*, which repressed *STC1* expression, but did not eliminate *STC1* expression (Fig. 7C, Additional file 1: Fig. S3). Repression of *STC1* did not inhibit early viral entry (Fig. 7A) based on measurement of a GFP reporter expressed by the KSHV virus or viral genomes per cell shortly after infection. STC1 knock-down led to a modest effect on the production of encapsidated viral genomes (average relative decrease of 29%, p = 0.0093) at 5 days post-infection in the conditions with the virus infections at a multiplicity of infection (MOI) at 0.5 (Fig. 7B). At 3-days post-infection, there was a mild reduction in expression of a KSHV latent gene, LANA, with siRNAs targeting *STC1* and a lower virus input (MOI 0.25) (Fig. 7C). The strongest effect with repression of *STC1* expression was found for the lytic switch gene, RTA, with an MOI of 0.25 for the KSHV infection. In sum, knocking down *STC1* modestly reduced viral gene expression and encapsidated viral genome production.

A hallmark feature of endothelial cell infections with KSHV is tubule formation. To test the impact of STC1 and FLT4 on angiogenesis in LECs during KSHV infection, tubule formation was measured. As expected, the tubule formation, as measured by the total length branched structures, increased with KSHV infection at 3 dpi (Fig. 7D). Notably, siRNAs targeting STC1 decreased the number of nodes, junctions, meshes and segments formed at 3 dpi (Fig. 7D). Targeting FLT4 with siRNA (Fig. 7D) also decreased the number of nodes, junctions, meshes and segments formed at 3 dpi for samples with confirmed FLT4 depletion at 2 dpi (Additional file 1: Fig. S3). Taken together, these results suggest that increased expression of STC1 or FLT4 may contribute to increased angiogenesis within KS lesions.

## Discussion

Kaposi sarcoma that manifests in the skin and GI tract leads to significant morbidity and mortality of individuals worldwide, especially among PWH. These analyses investigated similarities and differences in GI and skin KS lesions to discover host and viral factors associated with KS pathogenesis. To our knowledge, this is the first study evaluating and comparing KS lesions from different sites as well as the cellular and KSHV transcripts from KS tissues within a well annotated cohort from the United States. Heterogeneity was noted in the viral gene expression profile, but several canonical lytic genes were consistently detected irrespective of clinical characteristics. While we observed considerable heterogeneity in the expression of host cellular genes between GI and skin lesions as compared to their matched normal tissues, 26 common cellular genes of interest were identified between skin and GI lesions. These DEGs from KS lesions encode inflammatory cytokines as well as host factors associated with angiogenesis and immune regulation. Loss of function studies identified a role for STC1 and FLT4 in KSHV replication and virus-driven angiogenesis in KSHV-infected LECs.

We noted highly complex patterns of KSHV gene expression in these KS lesions that do not fit the canonical classifications of the latent versus lytic gene expression program in KS. There was higher expression of ORF72/v-cyclin, compared to ORF73/LANA in both skin and GI KS lesions. This observation was consistent with a report from a previous study that analyzed KSHV RNA expression from KS tumors and suggests transcription initiation from an alternative promoter called LTd [42]. The transcriptional regulation of promoter usage may differ between the KS lesions in this report and PEL cell lines. Detection of KSHV LANA protein is the standard assay for diagnosing KS. Several studies have previously reported expression of KSHV genes outside of the latency locus in KS samples from patients. In 2014, Hosseinipour et al. described a surprising amount of expression of K15 in multiple types of viral transcriptional programs [43]. K15, but not ORF75 expression was noted in this report using KS lesions from patient punch biopsies and measured with a qPCR array. Robust expression of ORF75 RNA was identified in all four patient KS skin biopsies in a study by Tso et al. using RNA sequencing [44]. This study also demonstrated higher expression of ORF75, than K15. It is important to recognize that K15 and ORF75 are adjacent genes and K15 is bicistronic [45]. A recent study also noted elevated ORF75 in three KS skin samples [46]. The expression analysis presented here suggests that if RNA expression techniques are utilized, additional KSHV genes may represent a more sensitive approach for detecting KS, potentially including ORF72 and ORF75. However, such technologies are limited by cost and analytical aspects in limited resource settings.

Overall, the participants had a low median CD4^+^ T cell count and HIV viremia was observed among those with GI KS. The heterogeneity between skin and GI samples may be related to the underlying clinical characteristics and concurrent KAD. Elevated inflammatory cytokines (IL-1, IL-6 and IL-10) are notably high in the circulation among individuals diagnosed with concurrent KSHV-associated diseases [3, 26, 27]. Interestingly, there were no differences in these key cytokine levels by presence of concurrent KAD in skin or GI KS. Skin KS had higher IL-6 and IL-10 gene expression compared to normal tissue, whereas GI KS had higher IL-1A expression. In skin KS lesions, IL-6 gene expression had a positive correlation with the sum of KSHV transcripts; this trend was not observed in GI lesions. This differential correlation may preliminarily infer the role of KSHV infection in regulating IL-6 expression specifically in skin KS lesions. The variation observed in a number of host DEGs in the GI KS as compared to the skin KS may be related to responses of tumor cells towards specific organs and the activation of specific genes, pathways and interaction with distinct immune surveillance mechanisms within these organs that can result in heterogenous expression by tumor site, which has been noted in other malignant conditions [47, 48].

A previous study of 4 participants with HIV and cutaneous KS identified cellular genes associated with lipid and glucose metabolism [44]; however, this was not observed in the current study. We observed several interferon genes that were increased in KS lesions. *IFNAR2* alone was elevated in the skin KS lesions as compared to the normal skin. A wide range of *IFNG* expression changes were observed in both skin and GI KS lesions as compared to the normal tissue. Among these tissues that were evaluated, it is interesting to note that among the KSHV genes, the viral interferon regulatory factors (vIRF) expression, which is known to regulate antiviral response, was variable in its expression (highest expression of vIRF4) in all tissues [49, 50]. In this small cohort, the differential expression of *CCBE1* may indicate the diagnosis of KS alone and the absence of concurrent active KAD. As a possible tumor suppressor gene [51], the decreased expression of *CCBE1* in those with concurrent KAD and KS (where KSHV viremia in the circulation is expected to be higher) requires further evaluation.

Among cellular genes in both GI and skin KS lesions as compared to their respective normal tissues, both FLT4 and STC1 were increased. FLT4 specifically has been noted in previous studies in HIV-associated KS [39, 44]. The significance of this study is the use of KSHV infected LECs to further investigate the role of both STC1 and FLT4 on KSHV infection in cell culture models. In exploring angiogenesis within LECs using tube formation assays, inhibition of STC1 and FLT4 led to a decrease in tubule formation. The increased expression of genes associated with angiogenic pathways raise the possibility of exploring additional anti-angiogenic therapies. Bevacizumab, a monoclonal antibody against VEGF-A, both alone [52] and in combination liposomal doxorubicin [53] led to modest responses, but the overall response rate was less as compared to other approved KS therapies. Newer drugs active against FLT4 or VEGFR3 have been recently licensed for use in medullary thyroid cancer [54] and may be a promising therapy to consider in KS. Other findings from this study with possible therapeutic potential include targeting for IL1A, such as anakinra, which has been shown to treat a wide range of inflammatory syndromes [55]. This may be useful as a possible combination therapy for patients who present with KS in the GI tract alone. Previous reports identified genes important for survival of KSHV-infected cells in B cells [56] and endothelial cells [57]. Some of these genes were upregulated in our analyses of skin KS (*BTAF, VMP1, DCHS1, VCAM1, PLXND1*) (Fig. S4). Additionally, the immune profiles seen in these analyses reinforce the use of chemotherapy sparing options, such as immunotherapy, which may increase T cell stimulation and reduce markers of T cell exhaustion associated with chronic HIV and KSHV infection [58].

The heterogenous clinical characteristics, such as the presence of concurrent KAD, prior treatment, KSHV levels and HIV characteristics, within this study are limitations that impact the generalizability of these findings. Despite these limitations, these findings provide a broad overview for subsequent analyses, specifically for GI KS that is associated with severe manifestations of this disease and remains rarely studied. Moreover, as there are no animal models for the study of KS, analyses of lesions from patients with KS provide important insights to factors and possible therapeutic targets that require further analyses.

In summary, this study evaluates a selection of participants from the United States presenting with skin and GI KS. These analyses highlight differences in the KS immune profiles, angiogenic factors and KSHV gene expression by site of involvement. Future studies may include investigating the spatial association of these gene expression patterns across numerous samples to better understand how KSHV-infected cells interact with the immune system and the tumor microenvironment to impact these changes.

### Supplementary Information

**Additional file 1: Table S1.** KSHV inflammatory cytokine syndrome case definition from Polizzotto MN, Uldrick TS, Wyvill KM, Aleman K, Marshall V, Wang V, et al. Clinical Features and Outcomes of Patients With Symptomatic Kaposi Sarcoma Herpesvirus (KSHV)-associated Inflammation: Prospective Characterization of KSHV Inflammatory Cytokine Syndrome (KICS). Clin Infect Dis. 2016;62(6):730-8. **Tables S2 and S3.** Pathway enrichment for differentially expressed genes (DEGs) in skin (S2) and GI (S3) samples. **Table S4.** Spearman correlation analysis between specific human gene expression and total KSHV gene expression. **Figure S1.** After RNA sequencing, gene expression profiles of combined human and viral genes were analyzed using principal component analysis for skin samples (A), GI samples (B), and combined (C). “N” at the end of sample names are the normal tissue samples. “T” in sample names refers KS tumor samples. D. KSHV transcript per million (TPM) values were combined for all KSHV genes. Stronger red backgrounds indicate higher KSHV expression. **Figure S2.** Human gene expression patterns in matched samples. A. Inflammatory and interferon genes associated with viral pathogenesis or immune responses are plotted. B. T cell genes associated with viral pathogenesis or immune responses are plotted. Asterisks (blue* for GI and orange * for skin) represent statistically significant (Student’s t test, p < 0.05) genes, in individual GI (tumor vs normal, log2 Fold Change, blue squares) and skin (tumor vs normal, log2 Fold Change, orange dots) samples. **Figure S3.** Induction of STC1 during infection and depletion with siRNAs. Induction of STC1 secreted protein (A) and transcript (B) for de novo infected HDLEC cells and corresponding reduction with siSTC1 in tube formation assay. Similarly, FLT4 transcript was depleted with siFLT4 (C). Samples shown as paired to respective controls (Mock siNeg, Inf siNeg). RT-qPCR was used to determine expression differences. D. Western blotting for STC1 and FLT4 (E) after KSHV infection and transfection with siRNAs. **Figure S4.** CRISPR screens previously reported and skin KS expression changes (tumor vs. normal). Depleted targets (blue dots) were hits from CRISPR screens for killing KSHV-infected cells in PEL cells (A) or endothelial cells (B). Red dots depict the average fold changes in RNA expression from skin KS lesions compared to normal skin (introduced in Fig. 1).

## Data Availability

Sequencing data are available at NCBI Gene Expression Omnibus accession number GSE241095.
