# Supplementary material for: Transcriptional landscape of Kaposi sarcoma tumors identifies unique immunologic signatures and key determinants of angiogenesis
Source: J Transl Med. 2023 Sep 22;21:653. doi: 10.1186/s12967-023-04517-5 (PMC10517594; doi:10.1186/s12967-023-04517-5)
Supplement: Supplementary file 1 — Additional file 1: Table S1. KSHV inflammatory cytokine syndrome case definition from Polizzotto MN, Uldrick TS, Wyvill KM, Aleman K, Marshall V, Wang V, et al. Clinical Features and Outcomes of Patients With Symptomatic Kaposi Sarcoma Herpesvirus (KSHV)-associated Inflammation: Prospective Characterization of KSHV Inflammatory Cytokine Syndrome (KICS). Clin Infect Dis. 2016;62(6):730-8. Tables S2 and S3. Pathway enrichment for differentially expressed genes (DEGs) in skin (S2) and GI (S3) samples. Table S4. Spearman correlation analysis between specific human gene expression and total KSHV gene expression. Figure S1. After RNA sequencing, gene expression profiles of combined human and viral genes were analyzed using principal component analysis for skin samples (A), GI samples (B), and combined (C). “N” at the end of sample names are the normal tissue samples. “T” in sample names refers KS tumor samples. D. KSHV transcript per million (TPM) values were combined for all KSHV genes. Stronger red backgrounds indicate higher KSHV expression. Figure S2. Human gene expression patterns in matched samples. A. Inflammatory and interferon genes associated with viral pathogenesis or immune responses are plotted. B. T cell genes associated with viral pathogenesis or immune responses are plotted. Asterisks (blue* for GI and orange * for skin) represent statistically significant (Student’s t test, p < 0.05) genes, in individual GI (tumor vs normal, log2 Fold Change, blue squares) and skin (tumor vs normal, log2 Fold Change, orange dots) samples. Figure S3. Induction of STC1 during infection and depletion with siRNAs. Induction of STC1 secreted protein (A) and transcript (B) for de novo infected HDLEC cells and corresponding reduction with siSTC1 in tube formation assay. Similarly, FLT4 transcript was depleted with siFLT4 (C). Samples shown as paired to respective controls (Mock siNeg, Inf siNeg). RT-qPCR was used to determine expression differences. D. Western blotting for STC1 [file 12967_2023_4517_MOESM1_ESM.pdf]

Supplementary Tables and Figures

**1. CLINICAL MANIFESTATIONS***	
**a. Symptoms**	**b. Laboratory abnormalities**
Fever	Anemia
Fatigue	Thrombocytopenia
Edema	Hypoalbuminemia
Cachexia	Hyponatremia
Respiratory symptoms (including cough, dyspnea, airway hyperreactivity)	**c. Radiographic abnormalities**
Gastrointestinal disturbance (including nausea, anorexia, abdominal discomfort, altered bowel habit)	Lymphadenopathy
Arthralgia and myalgia	Splenomegaly
Altered mental state	Hepatomegaly
Neuropathy with or without pain	Body cavity effusions
**2. EVIDENCE OF SYSTEMIC INFLAMMATION**	
Elevated C-reactive protein	
**3. EVIDENCE OF KSHV LYTIC ACTIVITY**	
Elevated KSHV viral load in peripheral blood mononuclear cells ($\geq$100 copies/10^6 cells)	
**4. NO EVIDENCE OF KSHV-ASSOCIATED MULTICENTRIC CASTLEMAN DISEASE**	
Exclusion of MCD requires pathologic assessment lymph node, bone marrow, or spleen	

The working case definition of KICS requires the presence of at least two clinical manifestations drawn from at least two categories (1a, b, and c), together with each of the criteria in 2, 3, and 4. Clinical manifestations for the working definition are drawn from the initial case series and from findings commonly seen in KSHV–MCD.

Table S1: KSHV inflammatory cytokine syndrome case definition from Polizzotto MN, Uldrick TS, Wyvill KM, Aleman K, Marshall V, Wang V, et al. Clinical Features and Outcomes of Patients With Symptomatic Kaposi Sarcoma Herpesvirus (KSHV)-associated Inflammation: Prospective Characterization of KSHV Inflammatory Cytokine Syndrome (KICS). Clin Infect Dis. 2016;62(6):730-8.

**Table S2. Canonical Pathways in skin DEGs**	**-log(p-value)**	**Ratio**	**z-score**
Hepatic Fibrosis / Hepatic Stellate Cell Activation	1.65E+01	1.24E-01	NaN
Systemic Lupus Erythematosus In B Cell Signaling Pathway	1.25E+01	5.12E-02	-0.333
IL-15 Signaling	1.06E+01	5.49E-02	NaN
Atherosclerosis Signaling	1.01E+01	1.15E-01	NaN
B Cell Receptor Signaling	8.79E+00	4.61E-02	NaN
Agranulocyte Adhesion and Diapedesis	8.75E+00	7.94E-02	NaN
Granulocyte Adhesion and Diapedesis	8.67E+00	8.47E-02	NaN
Communication between Innate and Adaptive Immune Cells	8.21E+00	3.77E-02	NaN
Tumor Microenvironment Pathway	7.22E+00	7.82E-02	3.606
GP6 Signaling Pathway	7.17E+00	9.45E-02	3.464
Inhibition of Matrix Metalloproteases	6.32E+00	1.79E-01	-1.89
Osteoarthritis Pathway	5.80E+00	5.98E-02	2.111
Axonal Guidance Signaling	5.20E+00	3.95E-02	NaN
Role of Macrophages, Fibroblasts and Endothelial Cells in Rheumatoid Arthritis	4.80E+00	4.62E-02	NaN
Hepatic Fibrosis Signaling Pathway	4.64E+00	4.06E-02	1.604
LXR/RXR Activation	4.61E+00	7.32E-02	0.378
Role of Osteoblasts, Osteoclasts and Chondrocytes in Rheumatoid Arthritis	4.58E+00	5.36E-02	NaN
Airway Pathology in Chronic Obstructive Pulmonary Disease	3.93E+00	6.78E-02	NaN
Glucocorticoid Receptor Signaling	3.86E+00	3.27E-02	NaN
Pathogenesis of Multiple Sclerosis	3.82E+00	3.33E-01	NaN
Neuroinflammation Signaling Pathway	3.75E+00	4.13E-02	2.714
HOTAIR Regulatory Pathway	3.67E+00	5.52E-02	2.828
Apelin Liver Signaling Pathway	3.55E+00	1.54E-01	2
Th1 and Th2 Activation Pathway	3.49E+00	5.23E-02	NaN
Phagosome Formation	3.36E+00	2.90E-02	4.472
Role of Cytokines in Mediating Communication between Immune Cells	3.25E+00	9.26E-02	NaN
Role of Hypercytokinemia/hyperchemokinemia in the Pathogenesis of Influenza	3.14E+00	6.98E-02	0
Role of Pattern Recognition Receptors in Recognition of Bacteria and Viruses	3.11E+00	5.13E-02	1
IL-6 Signaling	2.94E+00	5.47E-02	-0.378
HIF1A Signaling	2.90E+00	4.33E-02	3
LPS/IL-1 Mediated Inhibition of RXR Function	2.90E+00	3.98E-02	NaN
Intrinsic Prothrombin Activation Pathway	2.74E+00	9.52E-02	2
IL-10 Signaling	2.69E+00	6.94E-02	NaN
Acute Phase Response Signaling	2.64E+00	4.32E-02	0
IL-17 Signaling	2.61E+00	4.28E-02	0.707
Chondroitin Sulfate Biosynthesis (Late Stages)	2.56E+00	8.51E-02	1
Natural Killer Cell Signaling	2.44E+00	4.02E-02	-1.414
p38 MAPK Signaling	2.44E+00	5.08E-02	-0.816
Gustation Pathway	2.43E+00	4.00E-02	1.414
Th1 Pathway	2.37E+00	4.92E-02	1.342
IL-15 Production	2.35E+00	4.88E-02	2.449
Chondroitin Sulfate Biosynthesis	2.31E+00	7.27E-02	1
Dermatan Sulfate Biosynthesis	2.23E+00	6.90E-02	1
Colorectal Cancer Metastasis Signaling	2.16E+00	3.35E-02	2.828
STAT3 Pathway	2.15E+00	4.44E-02	NaN
Th2 Pathway	2.12E+00	4.38E-02	2.236
Œ≥-glutamyl Cycle	2.10E+00	1.82E-01	NaN
Airway Inflammation in Asthma	2.10E+00	9.09E-02	NaN
Iron homeostasis signaling pathway	2.10E+00	4.32E-02	NaN
Glutamate Receptor Signaling	2.03E+00	6.06E-02	NaN
Regulation Of The Epithelial Mesenchymal Transition By Growth Factors Pathway	1.98E+00	3.65E-02	2.646
Leukocyte Extravasation Signaling	1.97E+00	3.63E-02	1.89
Complement System	1.96E+00	8.11E-02	NaN
Notch Signaling	1.93E+00	7.89E-02	NaN
PI3K/AKT Signaling	1.90E+00	3.52E-02	NaN
Leukotriene Biosynthesis	1.89E+00	1.43E-01	NaN
Bladder Cancer Signaling	1.82E+00	4.31E-02	NaN
Oncostatin M Signaling	1.79E+00	6.98E-02	NaN
Dendritic Cell Maturation	1.79E+00	2.38E-02	2.138
Cardiac Hypertrophy Signaling (Enhanced)	1.74E+00	2.42E-02	1.414
Dermatan Sulfate Biosynthesis (Late Stages)	1.73E+00	6.67E-02	NaN
Differential Regulation of Cytokine Production in Macrophages and T Helper Cells by IL-17A and IL-17F	1.68E+00	1.11E-01	NaN
FXR/RXR Activation	1.68E+00	3.97E-02	NaN
Hepatic Cholestasis	1.50E+00	3.17E-02	NaN
CSDE1 Signaling Pathway	1.49E+00	5.36E-02	NaN
Differential Regulation of Cytokine Production in Intestinal Epithelial Cells by IL-17A and IL-17F	1.48E+00	8.70E-02	NaN
Apelin Cardiac Fibroblast Signaling Pathway	1.48E+00	8.70E-02	NaN
Synaptic Long Term Depression	1.45E+00	3.09E-02	1.633
L-serine Degradation	1.43E+00	3.33E-01	NaN
Neuropathic Pain Signaling In Dorsal Horn Neurons	1.42E+00	3.96E-02	1
CREB Signaling in Neurons	1.40E+00	2.16E-02	2.496
PTEN Signaling	1.40E+00	3.33E-02	NaN
Semaphorin Neuronal Repulsive Signaling Pathway	1.39E+00	3.31E-02	NaN
PD-1, PD-L1 cancer immunotherapy pathway	1.36E+00	3.77E-02	NaN
Sertoli Cell-Sertoli Cell Junction Signaling	1.35E+00	2.91E-02	NaN
Aryl Hydrocarbon Receptor Signaling	1.31E+00	3.14E-02	-1
Eicosanoid Signaling	1.29E+00	4.48E-02	NaN
Role of JAK1 and JAK3 in Œ≥c Cytokine Signaling	1.26E+00	4.35E-02	NaN
SPINK1 General Cancer Pathway	1.26E+00	4.35E-02	NaN
HMGB1 Signaling	1.23E+00	2.99E-02	NaN
Heparan Sulfate Biosynthesis (Late Stages)	1.23E+00	4.23E-02	NaN
Œ±-tocopherol Degradation	1.22E+00	2.00E-01	NaN
Citrulline-Nitric Oxide Cycle	1.22E+00	2.00E-01	NaN
Fatty Acid Œ≤-oxidation I	1.17E+00	5.88E-02	NaN
Caveolar-mediated Endocytosis Signaling	1.17E+00	4.00E-02	NaN
Role of MAPK Signaling in Inhibiting the Pathogenesis of Influenza	1.16E+00	3.95E-02	NaN
Xenobiotic Metabolism Signaling	1.16E+00	2.43E-02	NaN
Erythropoietin Signaling Pathway	1.15E+00	2.82E-02	0.447
TREM1 Signaling	1.15E+00	3.90E-02	NaN
Chondroitin and Dermatan Biosynthesis	1.14E+00	1.67E-01	NaN
Tryptophan Degradation to 2-amino-3-carboxymuconate Semialdehyde	1.14E+00	1.67E-01	NaN
Heparan Sulfate Biosynthesis	1.13E+00	3.85E-02	NaN
Toll-like Receptor Signaling	1.13E+00	3.85E-02	NaN
IL-17A Signaling in Fibroblasts	1.09E+00	5.26E-02	NaN
GABA Receptor Signaling	1.09E+00	3.05E-02	NaN
HGF Signaling	1.08E+00	3.03E-02	NaN
Xenobiotic Metabolism CAR Signaling Pathway	1.06E+00	2.66E-02	0.447
TR/RXR Activation	1.06E+00	3.57E-02	NaN
Xenobiotic Metabolism PXR Signaling Pathway	1.04E+00	2.62E-02	0.447
RAC Signaling	1.03E+00	2.90E-02	NaN
GŒ±i Signaling	1.03E+00	2.90E-02	1
Sperm Motility	1.00E+00	2.36E-02	NaN
Regulation of Cellular Mechanics by Calpain Protease	1.00E+00	3.37E-02	NaN
MIF Regulation of Innate Immunity	9.83E-01	4.55E-02	NaN
Retinol Biosynthesis	9.83E-01	4.55E-02	NaN
Adrenomedullin signaling pathway	9.83E-01	2.51E-02	-1
Role of IL-17F in Allergic Inflammatory Airway Diseases	9.67E-01	4.44E-02	NaN
Actin Nucleation by ARP-WASP Complex	9.59E-01	3.23E-02	NaN
iNOS Signaling	9.36E-01	4.26E-02	NaN
Triacylglycerol Biosynthesis	9.36E-01	4.26E-02	NaN
Glycine Betaine Degradation	9.28E-01	1.00E-01	NaN
Ketogenesis	8.89E-01	9.09E-02	NaN

Table S3. Canonical Pathways in GI DEGs	-log(p-value)	Ratio	z-score	DEGs
Granulocyte Adhesion and Diapedesis	7.00E+00	4.23E-02	NaN	CXCL5,CXCL8,IL1A,MMP12,MMP7,PPBP,SELE,TNFRSF11B
Agranulocyte Adhesion and Diapedesis	5.43E+00	3.27E-02	NaN	CXCL5,CXCL8,IL1A,MMP12,MMP7,PPBP,SELE
Role of IL-17A in Psoriasis	5.03E+00	2.14E-01	NaN	CXCL5,CXCL8,S100A9
Acute Phase Response Signaling	4.70E+00	3.24E-02	NaN	C4BPA,IL1A,SAA1,SAA2,SERPINA3,TNFRSF11B
Osteoarthritis Pathway	4.13E+00	2.56E-02	1.342	ACAN,ADAMTS4,CXCL8,H19,MMP12,S100A9
Role of Macrophages, Fibroblasts and Endothelial Cells in Rheumatoid Arthritis	3.36E+00	1.85E-02	NaN	ADAMTS4,CXCL8,FCGR3A/FCGR3B,IL1A,SELE,TNFRSF11B
LXR/RXR Activation	3.28E+00	3.25E-02	0	IL1A,SAA1,SAA2,TNFRSF11B
HMGB1 Signaling	2.79E+00	2.40E-02	2	CXCL8,IL1A,SELE,TNFRSF11B
IL-17 Signaling	2.61E+00	2.14E-02	1	CXCL5,CXCL8,IL1A,TNFRSF11B
Hepatic Fibrosis / Hepatic Stellate Cell Activation	2.55E+00	2.06E-02	NaN	CXCL8,FLT4,IL1A,TNFRSF11B
HIF1A Signaling	2.44E+00	1.92E-02	2	FLT4,MMP12,MMP7,NOX4
IL-8 Signaling	2.42E+00	1.90E-02	2	ANGPT2,CXCL8,FLT4,NOX4
EIF2 Signaling	2.33E+00	1.79E-02	NaN	MT-RNR1,MT-RNR2,NOX4,RNA5-8SN5
Bladder Cancer Signaling	2.28E+00	2.59E-02	NaN	CXCL8,MMP12,MMP7
Inhibition of Matrix Metalloproteases	2.21E+00	5.13E-02	NaN	MMP12,MMP7
FXR/RXR Activation	2.19E+00	2.38E-02	NaN	IL1A,SAA1,SAA2
IL-6 Signaling	2.17E+00	2.34E-02	NaN	CXCL8,IL1A,TNFRSF11B
Atherosclerosis Signaling	2.15E+00	2.31E-02	NaN	CXCL8,IL1A,SELE
STAT3 Pathway	2.10E+00	2.22E-02	NaN	FLT4,IL13RA2,IL1A
Glucocorticoid Receptor Signaling	2.09E+00	1.03E-02	NaN	CXCL8,IL13RA2,IL1A,KRT17,KRT7,SELE
Role of IL-17F in Allergic Inflammatory Airway Diseases	2.09E+00	4.44E-02	NaN	CXCL5,CXCL8
Hepatic Fibrosis Signaling Pathway	2.07E+00	1.19E-02	2.236	CXCL8,FLT4,IL1A,NOX4,TNFRSF11B
Role of Cytokines in Mediating Communication between Immune Cells	1.94E+00	3.70E-02	NaN	CXCL8,IL1A
Role of Pattern Recognition Receptors in Recognition of Bacteria and Viruses	1.93E+00	1.92E-02	NaN	CXCL8,IL1A,PTX3
Senescence Pathway	1.91E+00	1.35E-02	2	CXCL8,IL1A,SAA1,SAA2
Role of IL-17A in Arthritis	1.89E+00	3.51E-02	NaN	CXCL5,CXCL8
HOTAIR Regulatory Pathway	1.88E+00	1.84E-02	NaN	MMP12,MMP7,TWIST1
Tumor Microenvironment Pathway	1.77E+00	1.68E-02	NaN	CXCL8,MMP12,MMP7
Hepatic Cholestasis	1.71E+00	1.59E-02	NaN	CXCL8,IL1A,TNFRSF11B
Coronavirus Pathogenesis Pathway	1.63E+00	1.48E-02	NaN	CXCL8,MT-RNR1,MT-RNR2
Role of Hypercytokinemia/hyperchemokinemia in the Pathogenesis of Influenza	1.56E+00	2.33E-02	NaN	CXCL8,IL1A
Role of Osteoblasts, Osteoclasts and Chondrocytes in Rheumatoid Arthritis	1.52E+00	1.34E-02	NaN	ADAMTS4,IL1A,TNFRSF11B
PPAR Signaling	1.38E+00	1.87E-02	NaN	IL1A,TNFRSF11B
Chondroitin Sulfate Degradation (Metazoa)	1.33E+00	6.25E-02	NaN	CEMIP
Airway Pathology in Chronic Obstructive Pulmonary Disease	1.31E+00	1.69E-02	NaN	CXCL8,IL1A
Dermatan Sulfate Degradation (Metazoa)	1.30E+00	5.88E-02	NaN	CEMIP
Differential Regulation of Cytokine Production in Intestinal Epithelial Cells by IL-17A and IL-17F	1.17E+00	4.35E-02	NaN	IL1A
Apelin Cardiac Fibroblast Signaling Pathway	1.17E+00	4.35E-02	NaN	ANGPT2
Neuroinflammation Signaling Pathway	1.16E+00	9.52E-03	NaN	CXCL8,GABRD,NOX4
Tumoricidal Function of Hepatic Natural Killer Cells	1.16E+00	4.17E-02	NaN	LYVE1
IL-17A Signaling in Gastric Cells	1.12E+00	3.85E-02	NaN	CXCL8
Cardiac Hypertrophy Signaling (Enhanced)	1.10E+00	7.43E-03	2	CXCL8,IL13RA2,IL1A,TNFRSF11B
eNOS Signaling	1.08E+00	1.26E-02	NaN	AQP9,FLT4
Airway Inflammation in Asthma	1.02E+00	3.03E-02	NaN	CXCL8
Erythropoietin Signaling Pathway	1.00E+00	1.13E-02	NaN	CXCL8,IL1A
Regulation of eIF4 and p70S6K Signaling	9.96E-01	1.12E-02	NaN	MT-RNR1,MT-RNR2
Dendritic Cell Maturation	9.91E-01	6.79E-03	2	FCGR3A/FCGR3B,FSCN1,IL1A,TNFRSF11B
Complement System	9.75E-01	2.70E-02	NaN	C4BPA
IL-17A Signaling in Fibroblasts	9.67E-01	2.63E-02	NaN	CXCL5
Regulation Of The Epithelial Mesenchymal Transition By Growth Factors Pathway	9.43E-01	1.04E-02	NaN	TNFRSF11B,TWIST1
Leukocyte Extravasation Signaling	9.39E-01	1.04E-02	NaN	MMP12,MMP7
Oncostatin M Signaling	9.14E-01	2.33E-02	NaN	CHI3L1
mTOR Signaling	8.73E-01	9.43E-03	NaN	MT-RNR1,MT-RNR2
MYC Mediated Apoptosis Signaling	8.54E-01	2.00E-02	NaN	TNFRSF11B
FAT10 Cancer Signaling Pathway	8.54E-01	2.00E-02	NaN	TNFRSF11B
SPINK1 Pancreatic Cancer Pathway	7.80E-01	1.67E-02	NaN	MMP12
LPS/IL-1 Mediated Inhibition of RXR Function	7.57E-01	7.97E-03	NaN	IL1A,TNFRSF11B
Induction of Apoptosis by HIV1	7.50E-01	1.54E-02	NaN	TNFRSF11B
IL-17A Signaling in Airway Cells	7.38E-01	1.49E-02	NaN	CXCL5
Colorectal Cancer Metastasis Signaling	7.12E-01	7.43E-03	NaN	MMP12,MMP7
IL-10 Signaling	7.10E-01	1.39E-02	NaN	IL1A
Axonal Guidance Signaling	7.10E-01	5.93E-03	NaN	ADAMTS4,MMP12,MMP7
Role of MAPK Signaling in Inhibiting the Pathogenesis of Influenza	6.88E-01	1.32E-02	NaN	CXCL8
TREM1 Signaling	6.84E-01	1.30E-02	NaN	CXCL8
Angiopoietin Signaling	6.84E-01	1.30E-02	NaN	ANGPT2
Toll-like Receptor Signaling	6.78E-01	1.28E-02	NaN	IL1A
Thyroid Cancer Signaling	6.74E-01	1.27E-02	NaN	CXCL8
VEGF Family Ligand-Receptor Interactions	6.50E-01	1.19E-02	NaN	FLT4
Apelin Adipocyte Signaling Pathway	6.40E-01	1.16E-02	NaN	NOX4
Xenobiotic Metabolism AHR Signaling Pathway	6.36E-01	1.15E-02	NaN	IL1A
Regulation Of The Epithelial Mesenchymal Transition In Development Pathway	6.36E-01	1.15E-02	NaN	TWIST1
Ceramide Signaling	6.23E-01	1.11E-02	NaN	TNFRSF11B
Crosstalk between Dendritic Cells and Natural Killer Cells	6.20E-01	1.10E-02	NaN	FSCN1
NF-ŒʃB Signaling	6.09E-01	5.26E-03	NaN	FLT4,IL1A,TNFRSF11B

Tables S2 and S3. Pathway enrichment for differentially expressed genes (DEGs) in skin (S2) and GI (S3) samples. Enriched pathway names are shown in the first column. The second

column shows the p-value for enrichment in -log format. The Ratio column shows the ratio of DEGs versus number of genes in each pathway. The z-score is a prediction based on DEG changes as to whether the overall pathway would be activated (positive z-score) or repressed (negative z-score) ("NaN" denotes a Z-score could not be calculated). The DEG column shows the individual DEGs in each pathway.

Correlation of sum of TPM values for all KSHV genes vs human genes

GI samples

Gene	Correlation p-value	R squared value
CALR	0.51	0.04
FLT4	0.51	0.04
IFNG	0.07	0.20
IL1A	0.01	0.51
IL6	0.98	4.90E-05
IL6R	0.17	0.18
IL10	0.60	0.03
STC1	0.04	0.38

Skin samples

Gene	Correlation p-value	R squared value
CALR	0.84	0.01
FLT4	0.35	0.11
IFNG	0.60	0.04
IL1A	0.20	0.20
IL6	0.02	0.56
IL6R	0.63	0.03
IL10	0.13	0.27
STC1	0.04	0.47

Table S4. Spearman correlation analysis between specific human gene expression and total KSHV gene expression.

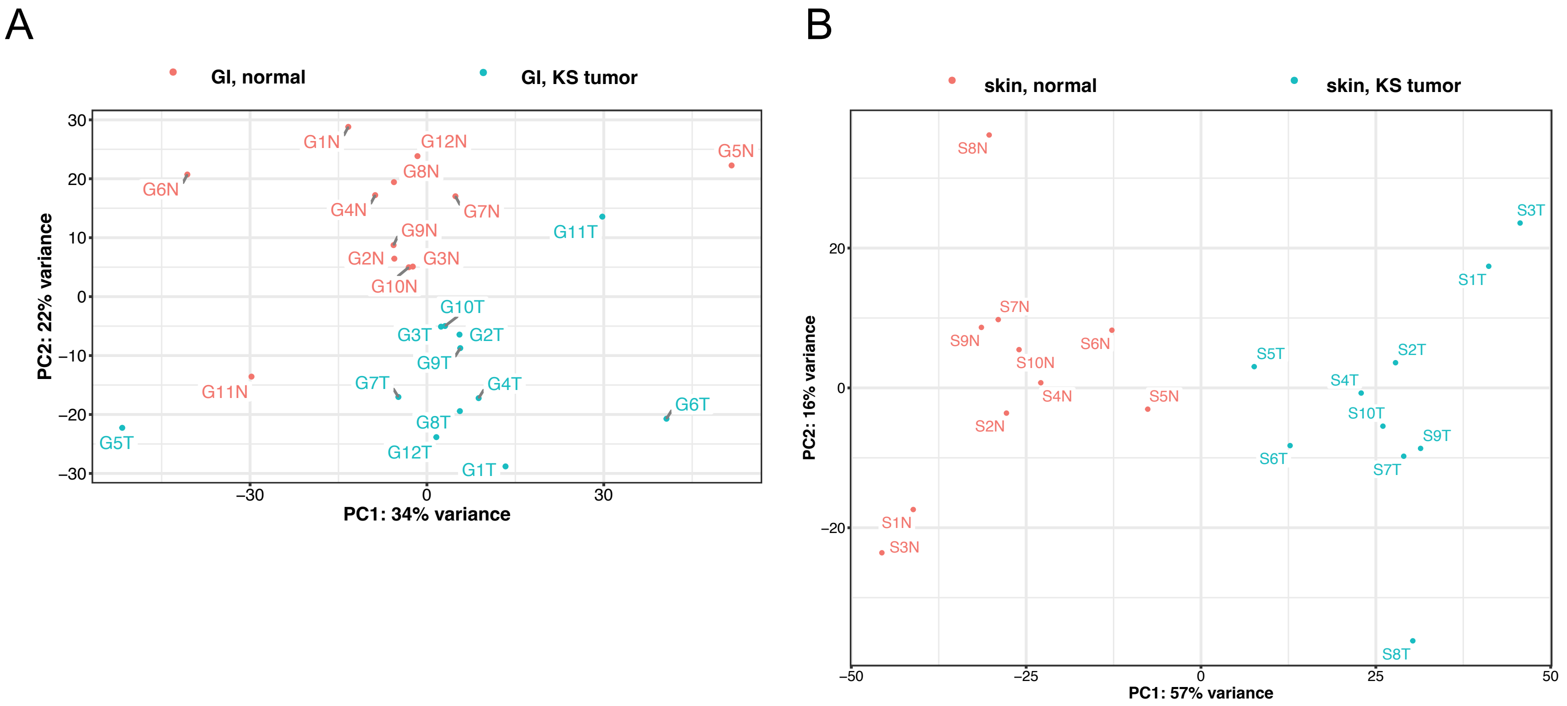

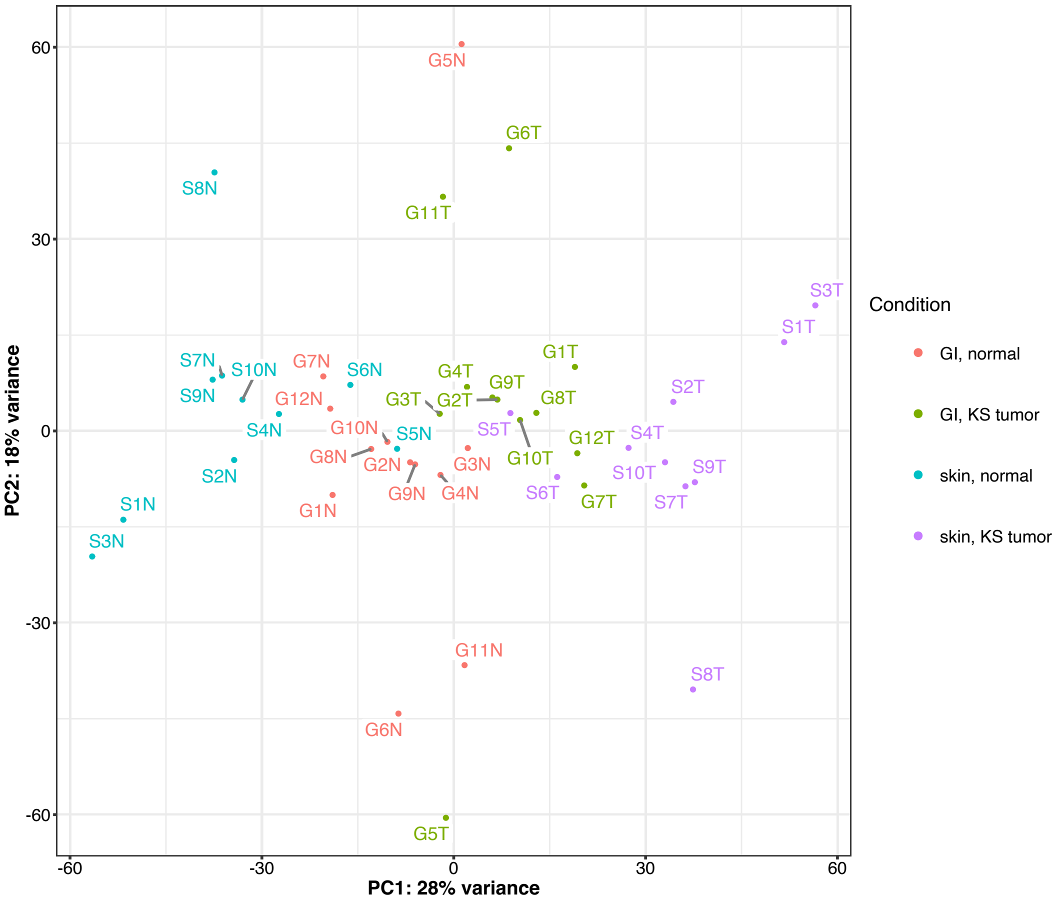

D

Tumor Sample	Total TPM values for KSHV genes
G1T	99.4
G2T	64.0
G3T	1.0
G4T	1.2
G5T	2.8
G6T	74.9
G7T	14.3
G8T	33.6
G9T	15.5
G10T	4.0
G11T	0.3
G12T	43.8
S1T	65.6
S2T	51.4
S3T	24.0
S4T	1.6
S5T	0.9
S6T	3.8
S7T	10.3
S8T	26.0
S9T	28.4
S10T	7.0

Figure S1: After RNA sequencing, gene expression profiles of combined human and viral genes were analyzed using principal component analysis for skin samples (A), GI samples (B), and combined (C). “N” at the end of sample names are the normal tissue samples. “T” in sample names refers KS tumor samples. D. KSHV transcript per million (TPM) values were combined for all KSHV genes. Stronger red backgrounds indicate higher KSHV expression.

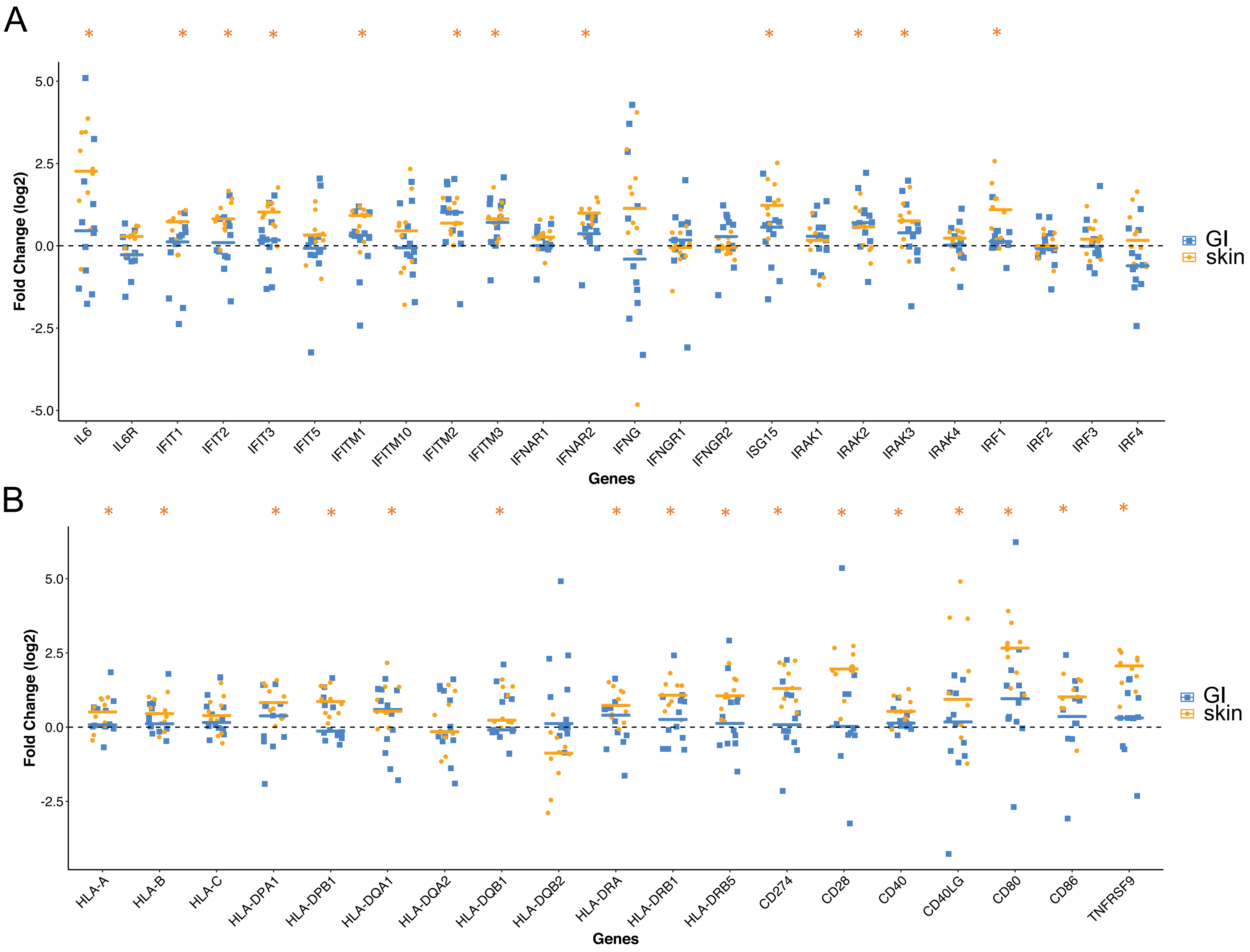

Figure S2: Human gene expression patterns in matched samples. A. Inflammatory and interferon genes associated with viral pathogenesis or immune responses are plotted. B. T cell genes associated with viral pathogenesis or immune responses are plotted. Asterisks (blue* for GI and orange * for skin) represent statistically significant (Student's t test, $p < 0.05$) genes, in individual GI (tumor vs normal, log2 Fold Change, blue squares) and skin (tumor vs normal, log2 Fold Change, orange dots) samples.

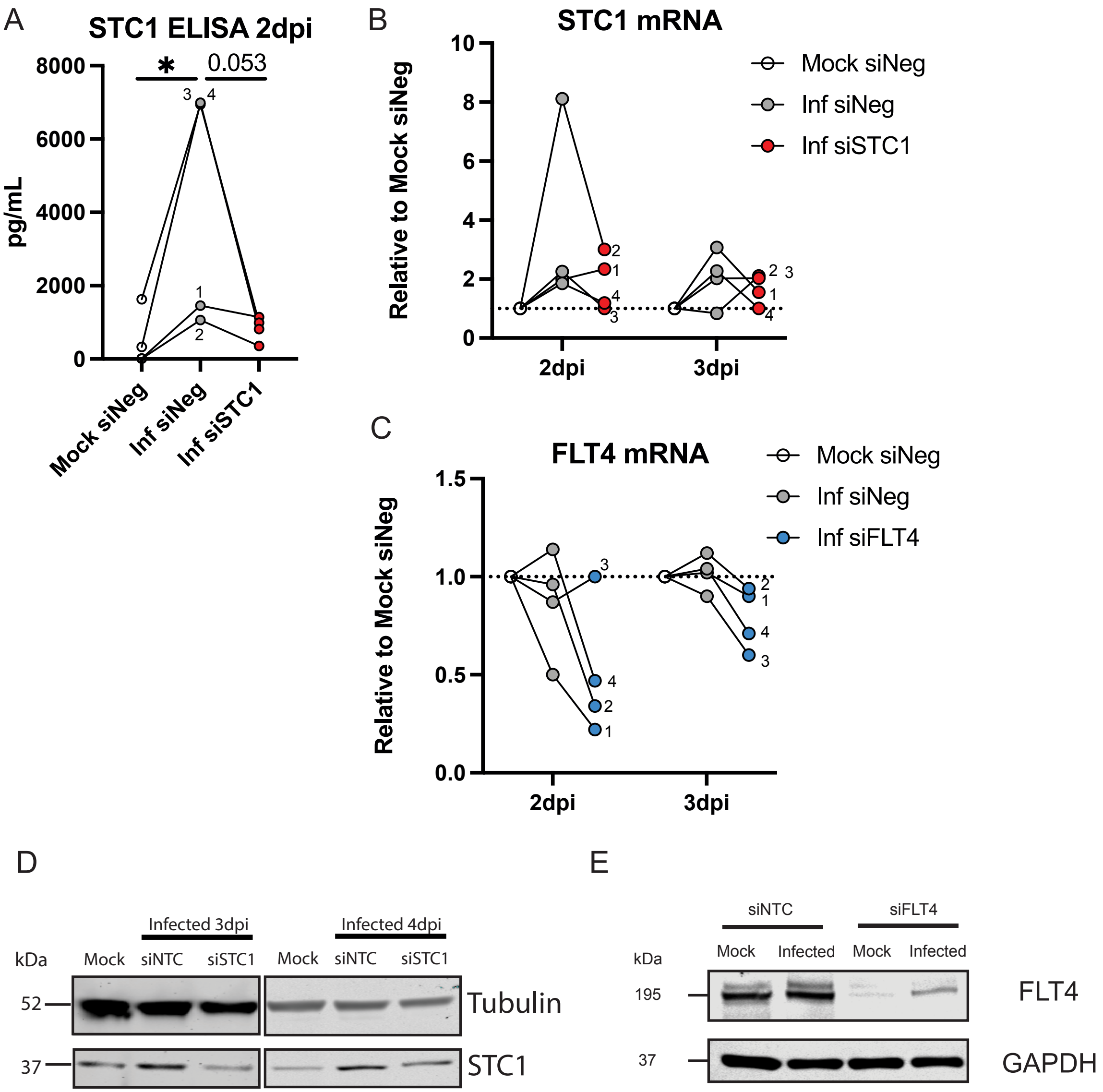

Figure S3. Induction of STC1 during infection and depletion with siRNAs. Induction of STC1 secreted protein (A) and transcript (B) for de novo infected HDLEC cells and corresponding reduction with siSTC1 in tube formation assay. Similarly, FLT4 transcript was depleted with siFLT4 (C). Samples shown as paired to respective controls (Mock siNeg, Inf siNeg). RT-qPCR was used to determine expression differences. D. Western blotting for STC1 and FLT4 (E) after KSHV infection and transfection with siRNAs.

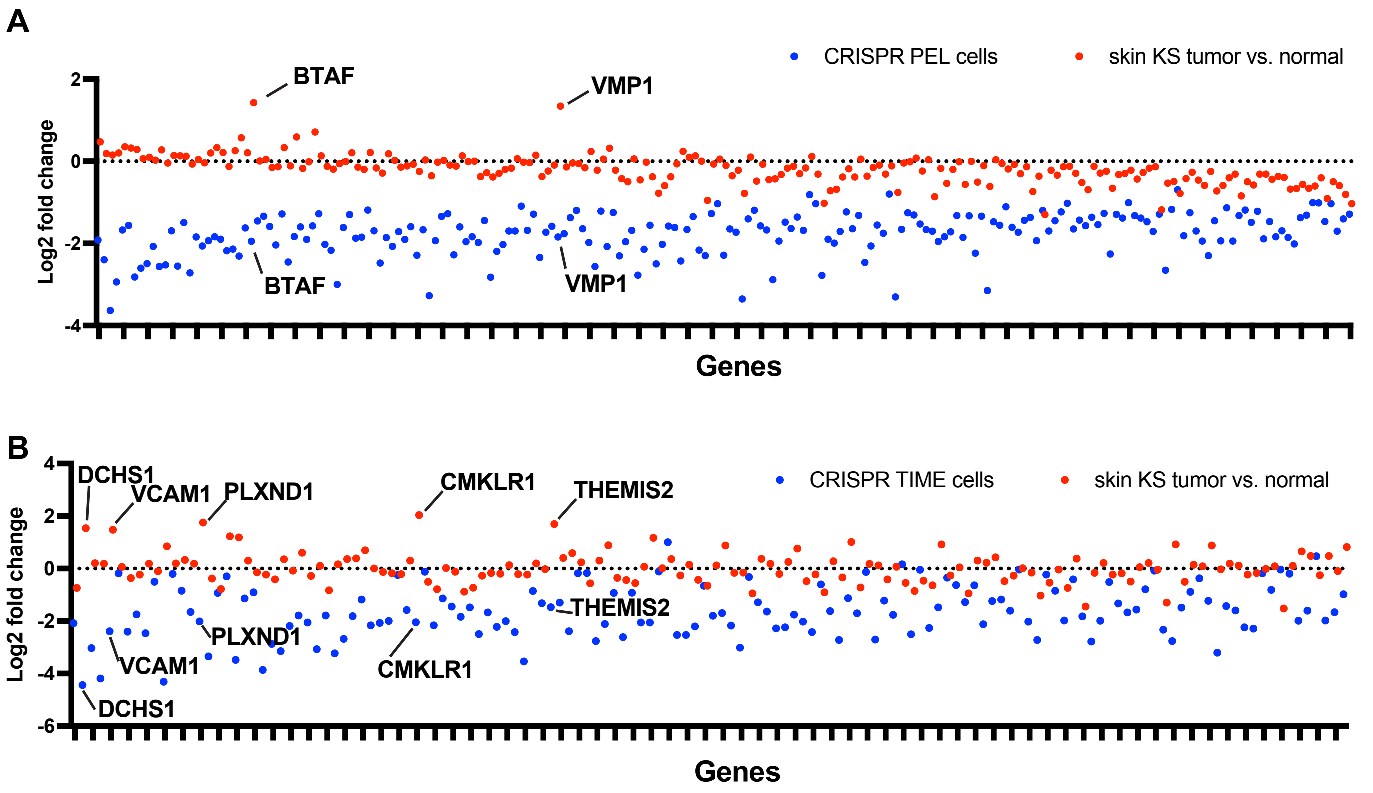

Figure S4. CRISPR screens previously reported and skin KS expression changes (tumor vs. normal). Depleted targets (blue dots) were hits from CRISPR screens for killing KSHV-infected cells in PEL cells (A) or endothelial cells (B). Red dots depict the average fold changes in RNA expression from skin KS lesions compared to normal skin (introduced in Fig. 1).
